# An epidemiological survey of COVID-19 serology and its association with clinical infection among older adults– does antibody titer matter?

**DOI:** 10.1186/s12877-024-04680-4

**Published:** 2024-02-15

**Authors:** Dvorah Sara Shapiro, Refael Ellis, Jowad Zidan, Yonit Wiener-Well, Maskit Bar-Meir, Eli Ben-Chetrit

**Affiliations:** 1 Department of Geriatrics, Shaare Zedek Medical Center, The Eisenberg R&D Authority, Hebrew University-Hadassah Medical School, Shmu’el Bait St 12, Jerusalem, Israel; 2 Infectious Diseases Unit, Shaare Zedek Medical Center, The Eisenberg R&D Authority, Hebrew University-Hadassah Medical School, Shmu’el Bait St 12, Jerusalem, Israel; 3 Pediatric Infectious Diseases Unit, Shaare Zedek Medical Center, The Eisenberg R&D Authority, Hebrew University-Hadassah Medical School, Shmu’el Bait St 12, Jerusalem, Israel

**Keywords:** COVID-19, Older adults, Infection rate, Antibody level

## Abstract

**Background:**

Older adults are at increased risk of severe SARS-CoV-2 infection. In this study we assessed the response to COVID-19 vaccination and infection rates among nursing homes (NH) and assisted-living care home (ALCH) residents.

**Methods:**

The study was conducted between August 2021 and January 2022, after widespread population vaccination with the third dose of Pfizer-BioNtech mRNA COVID-19 vaccine in Israel. Three groups were addressed: hospitalized older patients; NH and ALCH residents. Demographic data, COVID-19 serology (anti-spike IgG antibodies) and PCR test results were obtained to assess the dynamics of antibody titers and its correlation to infection rates.

**Results:**

Two-hundred eighty-five individuals were evaluated; 92 hospitalized patients; 100 ALCH residents and 93 NH residents. In the latter two groups two serology surveys were conducted three months apart. Hospitalized patients were younger than ALCH and NH residents (mean age 80.4 ± 8 versus 82.6 ± 8 and 83.6 ± 5, respectively, *p* = 0.01), and had more comorbidities (*p* = 0.003). The degree of decline in the antibody level overtime was similar in ALCH and NH residents. Infection rates were higher among NH residents than ALCH residents [35/91 (38.4%) versus 11/100 (11%), *p* < 0.001]. Antibody level was lower among those infected [2113 (1271–3512) Au/ml versus 4113 (3364–5029) Au/ml, *p* < 0.001]. Adjusted analysis showed that NH residence, but *not* antibody levels, were significantly associated with infection.

**Conclusion:**

Among older adults, infection rates inversely correlated with antibody level. However, only nursing home residence was significantly associated with infection, suggesting that other factors such as crowding considerably contribute to the risk of infection.

## Introduction

Old adults are at increased risk of developing severe disease after exposure to SARS-CoV-2 infection. The risk of hospitalization and death increases with age. Therefore, this population was the first to be vaccinated once the vaccine was available. Aging is associated with impaired capability to produce protective antibodies in response to immunization, resulting in reduced efficacy of vaccination [1, 2]. This may be partly due to impaired interactions between T and B cells which negatively influence the humoral response [3]. For example, the immune response after influenza vaccination, as measured by the hemagglutination inhibition assay-HAI, is weaker among older versus younger subjects receiving the same vaccination (4–5). Furthermore, frailty in older adults is characterized by a paucity of reserves in response to stress and is associated with low levels of IL-6 which correlate with poor response to Influenza vaccination [6].

The question thus arises regarding the immune response of older people after exposure to COVID-19 infection and vaccination. The aim of this study was to test the antibody response to COVID-19 vaccination among individuals aged 65 or older as well as infection rates during the *Omicron* wave.

## Methods

The study was performed between August 2021 and January 2022, after the approval of the third dose of Pfizer-BioNtech mRNA COVID-19 vaccine in Israel. Three population groups were addressed: the first included hospitalized patients in the medical/geriatric departments at the Shaare Zedek Medical Center (SZMC), Jerusalem, Israel. The second group comprised of adult individuals who reside in a nursing home (NH) (Neveh Horim, Nofey Yerushalaim, Jerusalem). The third group comprised of old adults residing in assisted-living care home (ALCH), (Ahuzat Beit Hakerem, and Nofey Yerushalaim, Jerusalem). Demographic and clinical data including age, gender, vaccination status, and comorbidities were collected using a structured questionnaire. Patients who were treated with active chemotherapeutic agents or high-dose corticosteroids (prednisone-equivalent ≥ 20 mg/day) were not included in the study.

COVID-19 serology (anti-spike IgG antibodies), (Abbott ARCHITECT SARS-CoV-2 IgG immunoassay, Abbott Park, IL) was tested at two timeframes: between Aug 2021 and Oct 2021 and during Dec 2021. Tests were performed at the central laboratory at SZMC.

During the study period, NH and ALCH residents were screened weekly for COVID-19 using PCR-based nasopharyngeal swabs (Seegene, Seoul, South Korea) as part of the Israeli Ministry of Health (MOH) policy to identify and isolate early infected patients. The tests were performed in two reference labs in Hadassah School of Medicine (Jerusalem) and Sheba Medical Center (Ramat Gan). The PCR results were obtained and were correlated to infection rates.

Analysis was performed initially for all study participants (immunized and recovered). Subsequent analysis was limited to individuals who were vaccinated three times (with no history of previous infection), thus allowing to examine the vaccine effect by itself.

The study was conducted as part of the Israeli MOH initiative for studies on COVID- 19 (no. 23) and was approved by the SZMC Institutional Review Board (approval no. 0063 − 21 SZMC).

### Statistical analysis

Continuous variables were presented as mean ± standard deviation (SD) or as median with interquartile range (IQR), as appropriate. Categorical variables were presented as numbers and percent. Categorical variables were compared between the study groups using Chi-square test. T-test or one-way ANOVA were used for normally distributed variables. Comparisons of continuous variables with non-normal distribution was performed using the Mann-Whitney U test (Wilcoxon Rank Sum Test). The association between residence (nursing home vs. independent assisted living) and the antibody titer was assessed with a linear regression model. Through this model, correction was made for the timing of the antibody test.

Adjusted analysis (using logistic regression) for variables which differed significantly between the study groups in the univariate analysis was used to assess which risk factor was likely associated with infection. A two-tailed *p* value of < 0.05 was considered statistically significant. Statistical analyses were performed using Epi-Info software, version 7.2 and SPSS version 25.

## Results

The study was carried out between August 2021 and January 2022. Overall, 285 participants were included. Ninety-two patients were hospitalized in the medical and geriatric departments at SZMC. One hundred ALCH residents and 93 NH residents were recruited.

Baseline demographic and clinical characteristics are presented in Table 1. The mean age of all study participants was 82 ± 7. ALCH residents were older than NH and hospitalized patients (*p* = 0.01, Table 1). Female patients comprised 63% of the cohort (*n* = 180). Hospitalized patients had significantly more comorbidities as compared with ALCH and NH residents (mean number of comorbidities 2.5 ± 1.4 versus 1.8 ± 1 versus 1.5 ± 1, respectively, *p* < 0.001).

**Table 1:** Demographic and clinical characteristics among the study groups

	All patients *n* = 285	Hospitalized patients, *n* = 92	Nursing home residents, *n* = 93	Assisted-living care home residents, *n* = 100	*P* value*
Age, mean ± SD	82 ± 7	80.4 ± 8	82.6 ± 8	83.6 ± 5	0.01
Gender (female), n (%)	180 (63)	48 (52)	60 (65)	72 (72)	0.01
Comorbidities n (%)					
Diabetes	98 (34.4)	53 (57.6)	29 (31.2)	16 (16)	< 0.001
Hypertension	194 (68)	64 (70)	65 (70)	65 (65)	0.7
Obesity	27 (9.5)	16 (17.4)	4 (4.3)	7 (7)	0.006
Cancer	38 (13)	14 (15.2)	11 (11.8)	13 (13)	0.8
Lung disease	45 (15.8)	21 (22.8)	14 (15)	10 (10)	0.05
Ischemic heart disease	141 (49.5)	59 (64.1)	42 (45.2)	40 (40)	0.002
Average(± SD) number of comorbidities	1.9 ± 1.2	2.5 ± 1.4	1.8 ± 1	1.5 ± 1	< 0.001

*Reference: hospitalized patients’ group

Most study participants (254/285) received the first COVID-19 vaccination (mRNA monovalent COVID-19 vaccine, Pfizer-BioNTech) between the end of December and the beginning of February 2020. The second vaccine dose was given 3–4 weeks later. The third vaccine was administered in most subjects (197/212) during August 2021. Two-hundred and three individuals were immunized with three vaccine doses (Table 2). Thirty-nine participants were previously infected (hospitalized patients, *n* = 10, NH residents, *n* = 21, and ALCH residents, *n* = 8).

**Table 2:** Geometric mean antibody titer (GMAT) of anti-spike IgG antibodies among the study groups

All patients, *n* = 285	Hospitalized patients (*n* = 92)	Nursing home residents (*n* = 93)	Assisted-living carehome residents (*n* = 100)	p value
1st GMAT Au/ml (95% CI)	1307 (858–1992)	5292 (3949–7093)	10,398 (8085–13,373)	< 0.0001
2nd GMAT Au/ml (95% CI)^a^	NA	3096 (2311–4148)	3738 (2899–4822)	0.68
Three-dose vaccine recipients^b^	41/92 (45%)	70/93 (76%)	91/100 (100%)	
1st GMAT Au/ml (95% CI) post 3rd vaccine	n = 41 3794 (2279–6319)	n = 70 6096 (4466–8321)	n = 91 11,367 (8845–14,608)	< 0.001
2nd GMAT Au/ml (95% CI) post 3rd vaccine^c^	NA	n = 68/70 3280 (2390–4502)	n = 91/100 3661 (2840–4719)	0.48

^a^No data was available for hospitalized patients

^b^COVID19-recovered patients were excluded (NH residents *n* = 21, ALCH residents, *n* = 8). Two additional NH residents and one ALCH resident were vaccinated twice and therefore excluded

^c^Two NH residents died before testing

The first serology test was sampled at a median of 51 (IQR 36–77) days after the 3rd vaccine dose. The second serology test was performed at a median of 141 (IQR 137–144) days after the 3rd vaccine dose and was available only in the ALCH and NH residents. Table 2 shows the anti-spike IgG antibody levels, presented as geometric mean antibody titer (GMAT, Au/ml) during the two timeframes, in the different study groups. Of note, in the nursing-home, serum samples during the first timeframe, were taken a month later than in ALCHs, with respect to the last vaccine dose. The second serology test samples were taken at both locations at the same time interval with respect to the last vaccine dose.

The anti-spike IgG antibody levels (GMAT, 95% CI) during the first timeframe were significantly higher among ALCH residents versus NH and hospitalized subjects (*p* < 0.0001) (Table 2). The results of the second serology test of NH and ALCH residents were comparable [3096 (2311–4148) versus 3738 (2899–4822), respectively (*p* = 0.68)].

Examining the anti-spike IgG antibody levels among three-dose vaccine recipients (excluding recovered patients), showed a similar trend (Table 2).

A sharper decrease was observed overtime in the antibody levels among patients residing in ALCH versus NH residents between the two serology tests that might have been attributed to the delay in obtaining serology tests in the NH residents (versus ALCH residents). To account for this delay (that may affect serology test results), a linear regression model was constructed with the difference between the two serology tests as the dependent variable, and the site of residence as the independent variable. A significant correlation was found between the site of residence and the decrease in the antibody titer (β=-0.3, *p* = 0.0001). However, after adjusting for the time variables (days between the third vaccination to the first serology test, e.g. first GMAT result, and days between the two measured titers) no difference was found in antibody titers decrease between the two patient groups (*p* = 0.2).

**Fig. 1:**
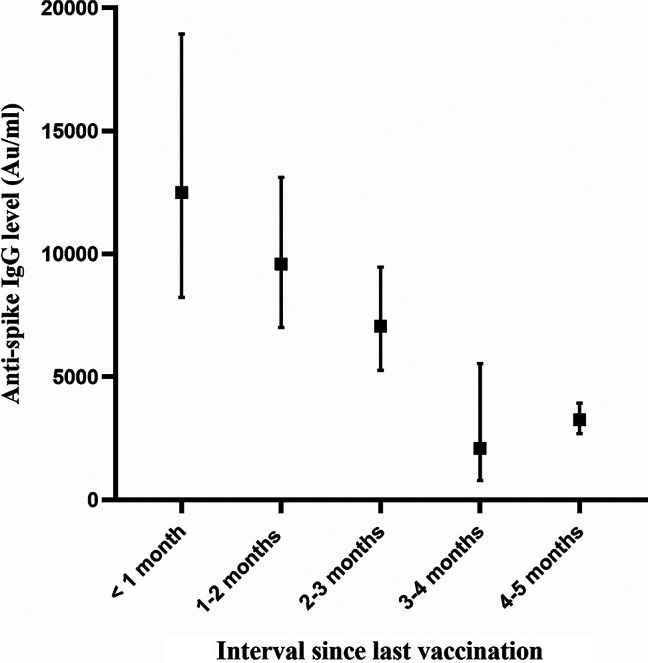
Change in SARS-CoV-2 anti-spike IgG (GMAT, 95% confidence interval) over a 5-month period among three-dose vaccine recipients

The change in COVID-19 anti-spike IgG among all study subjects who were vaccinated with three vaccine doses with respect to the time-interval since last vaccination is shown in Fig. 1. A decrease in antibody levels was observed over time.

Overall, 46 study participants were infected with COVID-19 during the study period. All infections occurred during January 2022. Of note, during January, a 4th vaccine dose was administered to most study subjects (183/191, 96%) in both, NH and ALCHs. Infections occurred within a median of 8 days (IQR 7–14) since the 4th vaccine dose. Table 3 summarizes demographic, clinical and serology test results among non-infected and infected subjects residing in the nursing-home and ALCHs. The rate of infection was higher among NH residents as compared to ALCH residents (35/91, 38.4% versus 11/100, 11%, respectively, *p* < 0.001). Low anti-spike IgG antibody levels were also associated with COVID-19 infection in a univariate analysis.

**Table 3:** Demographic, clinical and geometric mean antibody titers (GMAT) of anti-spike IgG antibodies among infected and non-infected subjects

	Non-infected	Infected	p value
All patients (n = 191)^a^	145	46	
Age, median (IQR)	84 (80–87)	85 (81–91)	0.38
Gender (female), n (%)	102 (70)	28 (61)	0.28
Residence			
Nursing home^b^ Assisted-living care homes	55 (61) 89 (89)	35 (38.9) 11 (11)	< 0.001
Diabetes	31 (21.4)	13 (28.3)	0.32
Hypertension	98 (67.6)	31 (67.4)	1
Obesity	6 (4.1)	5 (10.9)	0.14
Cancer	17 (11.7)	6 (13.3)	0.8
Lung disease	16 (11)	7 (15.2)	0.4
Ischemic heart disease	59 (40.7)	20 (43.5)	0.8
Number of comorbidities (mean ± SD)	2 ± 1.3	2.3 ± 1.3	0.1
Number of vaccines			
1 2 3	2 (1.3) 14 (10) 129 (89)	0 7 (15.2) 38 (85)	1 0.3 0.4
Past infection (recovered)	22 (15)	7 (15)	1
2nd antibody titer level (Geometric mean, 95% CI, Au/ml)	4468 (3629–5503)	2030 (1323–3115)	< 0.001

^a^Data unavailable, NH residents, *n* = 2

^b^One NH resident died (excluded)

**Fig. 2:**
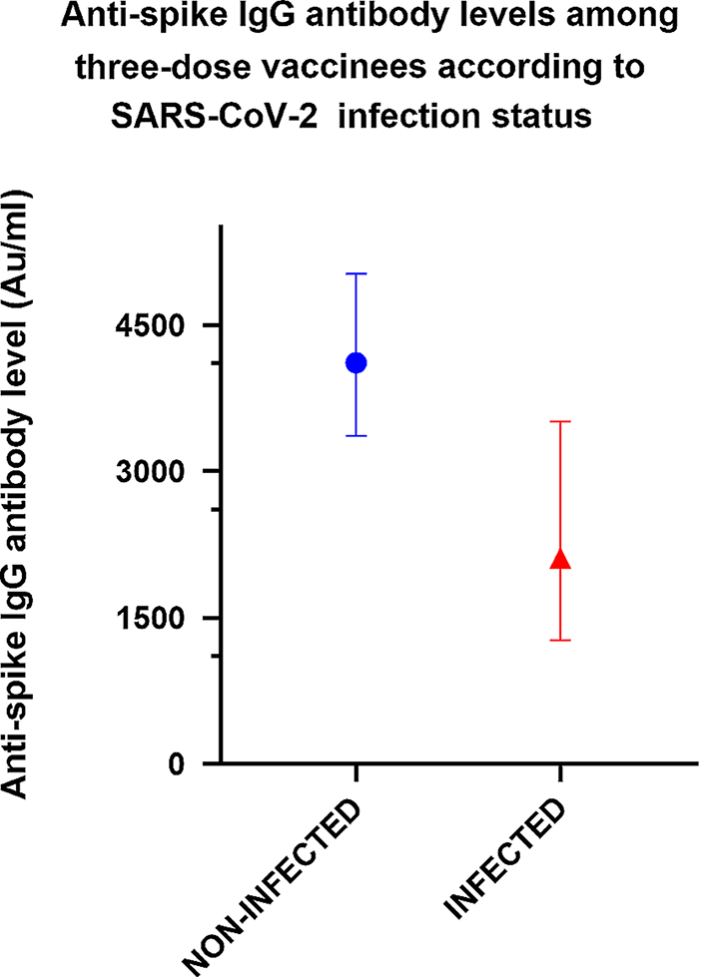
Anti-spike IgG antibodies (GMAT, 95% CI) among non-infected and infected three-dose vaccine recipients. GMAT of non-infected subjects (*n* = 120) was 4113 Au/ml (95% CI 3364–5029) versus 2113 Au/ml (95% CI 1271–3512) (infected individuals, *n* = 39), *p* = 0.01. COVID-19-recovered patients were excluded from analysis. Infection was diagnosed based on PCR testing

Figure 2 shows the anti-spike IgG antibody titer (GMAT, 95% CI) among non-infected versus infected subjects who received three vaccine doses (2nd titer post 3rd vaccination). The median (IQR) time (days) from last vaccination to infection was 162 days (IQR 161–168). Infected subjects had a significantly lower level of anti-spike IgG antibodies [4113 Au/ml (95% CI 3364–5029) versus 2113 Au/ml (95% CI 1271–3512), *p* = 0.01].

Administration of the 4th dose had no (short term) effect on the rate of infection; Infection occurred in 45/183 (24.6%) four-dose vaccinees versus 1/8 (12.5%) three-dose vaccinees (*p* = 0.7).

In an adjusted analysis (not shown), infection was significantly associated with residence in a nursing home [OR 6.4, 95% CI 2.7–15), *p* < 0.0001] but not anti-spike IgG levels (*p* = 0.3). Subsequent analysis including only three-dose vaccinees yielded similar results.

## Discussion

This study examined the humoral response (antibody levels) to COVID-19 vaccination in three groups of older adults and showed a rapid decrease in antibody level over a few months after vaccination in all study participants. This finding is supported by previous reports [7–9]. Lai et al. showed a decrease in the median antibody titer between 2 and 6 months after the second COVID-19 vaccination [7]. This has also been reported post infection [10]. Our study adds to the current literature that the decrease in anti-spike IgG levels continues also after a third dose (“first booster”) of the vaccine (Fig. 1).

Low anti-spike IgG levels apparently correlated with higher infection rates (*Omicron* variant) among vaccinated older individuals (Fig. 2). This has been shown in a study of young healthcare workers where antibody levels among *Omicron* variant-infected versus non-infected personnel were lower [11].

However, in the present study, subsequent analysis adjusting for residence, showed that the impact of antibody levels on infection rate was insignificant. This finding is supported by previous observation. In a study by Toress et al. no difference was noted in the antibody level between *Omicron variant-*infected and non-infected elderly individuals (average age 80 years) residing in a NH [12]. All subjects were immunized with three vaccine doses. Similar results were reported by Smoot et al., where no significant association was found between antibody level and infection observed during the *Omicron* surge [13].

This observation may be attributed, in part, to the unique characteristics of the *Omicron* variant (immune evasion, increased transmissibility, altered pathogenicity) as opposed to the *Delta* variant, where high antibody levels were significantly protective against infection [14].

Finally, nursing home residence was significantly associated with higher infection rates (despite no difference in the antibody levels among NH and ALCH residents). This may be attributed to several factors. The living conditions are more crowded in the nursing home compared to ALCHs. In nursing homes there are shared bedrooms, dining rooms and higher dependence on staff who move between patients– all of which may lead to spread of infections. Crowded nursing institutions were more likely to experience larger and deadlier COVID-19 outbreaks [15]. In contrast, ALCH residents have their own apartment and whilst there are communal activities (i.e., eating), during the COVID-19 outbreak, those were stopped.

In addition, although the number of comorbidities among infected NH and ALCH residents did not differ significantly, it is likely that NH residents may have been frailer with decreased functional capacity as compared to ALCH residents. Frailty has been associated with increased infection and mortality [16].

Last, other components of immunity were not tested in this study. For example, neutralizing antibody activity against SARS-CoV-2, IgA levels in the mucous membranes, as well as the specific response of CD4 and CD8 cells after illness or vaccination, to which long-term immune memory is attributed, were not evaluated [17–19]. All of which may have affected the risk of infection.

Important limitations of this study are its small sample size and the short-term follow-up of study participants regarding infection rate (one month post last serology test). The strengths of the study include the comparison of three different older population groups, with a longer follow up in the NH and ALCH groups, perhaps providing insight to the contribution of the third COVID-19-vaccine dose among older individual regarding both, anti-spike IgG dynamics and risk of infection.

In conclusion, we showed a significant decrease in the level of anti-spike IgG antibodies overtime among *three-dose* older adult vaccinees. Adjusted analysis showed that residing in a nursing home, rather than elevated antibody levels, was significantly associated with infection during the *Omicron* surge.

## Data Availability

The datasets used and/or analyzed during the current study are available from the corresponding author on reasonable request.

## Abbreviations

NH: nursing homes
ALCH: assisted-living care home
SZMC: Shaare Zedek Medical Center
MOH: Ministry of health

## References

[1] Bajaj V, Gadi N, Spihlman AP, Wu SC, Choi CH, Moulton VR (2020). Aging, immunity, and COVID-19: how Age influences the host Immune Response to Coronavirus infections?. Front Physiol.

[2] Dowery R, Benhamou D, Benchetrit E, Harel O, Nevelsky A, Zisman-Rozen S (2021). Peripheral B cells repress B-cell regeneration in aging through a TNF-α/IGFBP-1/IGF-1 immune-endocrine axis. Blood.

[3] Haynes L, Eaton SM (2005). The effect of age on the cognate function of CD4 + T cells. Immunol Rev.

[4] Goodwin K, Viboud C, Simonsen L (2006). Antibody response to influenza vaccination in the elderly: a quantitative review. Vaccine.

[5] Pinti M, Appay V, Campisi J, Frasca D, Fülöp T, Sauce D (2016). Aging of the immune system: focus on inflammation and vaccination. Eur J Immunol.

[6] Leng SX, Cappola AR, Andersen RE, Blackman MR, Koenig K, Blair M (2004). Serum levels of insulin-like growth factor-I (IGF-I) and dehydroepiandrosterone sulfate (DHEA-S), and their relationships with serum interleukin-6, in the geriatric syndrome of frailty. Aging Clin Exp Res.

[7] Lai A, Caimi B, Franzetti M, Bergna A, Velleca R, Gatti A et al. Durability of humoral responses after the second dose of mRNA BNT162b2 vaccine in residents of a Long Term Care Facility. Vaccines (Basel). 2022;10(3). 10.3390/vaccines10030446 PMC8954729 35335078

[8] Montejano-Hervás P, Gómez-Pavón J, Tornero-Torres O, Valverde-Moyar MV, Martín Cruz B, Vela Carbonera M (2022). Safety, Effectiveness, and immunogenicity 6 months after BNT162B2 mRNA vaccine in frail nursing home residents. Drugs Aging.

[9] Meyer M, Constancias F, Worth C, Meyer A, Muller M, Boussuge A (2022). Humoral immune response after COVID-19 infection or BNT162b2 vaccine among older adults: evolution over time and protective thresholds. Geroscience.

[10] Doke P, Gothankar JS, Doke PP, Kulkarni MM, Khalate KK, Shrivastava S (2022). Time dependent decline of neutralizing antibody titers in COVID-19 patients from Pune, India and evidence of reinfection. Microbes Infect.

[11] Möhlendick B, Čiučiulkaitė I, Elsner C, Anastasiou OE, Trilling M, Wagner B (2022). Individuals with weaker antibody responses after booster immunization are prone to omicron breakthrough infections. Front Immunol.

[12] Torres I, Giménez E, Albert E, Zulaica J, Álvarez-Rodríguez B, Burgos JS (2022). SARS-CoV-2 Omicron BA.1 variant breakthrough infections in nursing home residents after an homologous third dose of the Comirnaty® COVID-19 vaccine: looking for correlates of protection. J Med Virol.

[13] Smoot K, Yang J, Tacker DH, Welch S, Khodaverdi M, Kimble W (2022). Persistence and protective potential of SARS-CoV-2 antibody levels after COVID-19 vaccination in a West Virginia nursing home cohort. JAMA Netw Open.

[14] Willett BJ, Grove J, MacLean OA, Wilkie C, De Lorenzo G, Furnon W (2022). SARS-CoV-2 Omicron is an immune escape variant with an altered cell entry pathway. Nat Microbiol.

[15] Brown KA, Jones A, Daneman N, Chan AK, Schwartz KL, Garber GE (2021). Association between nursing home crowding and COVID-19 infection and mortality in Ontario, Canada. JAMA Intern Med.

[16] Fernandes AL, Pereira RMR (2022). Frailty in the context of COVID-19 pandemic: a life-threatening condition. Front Med (Lausanne).

[17] Khoury DS, Cromer D, Reynaldi A, Schlub TE, Wheatley AK, Juno JA (2021). Neutralizing antibody levels are highly predictive of immune protection from symptomatic SARS-CoV-2 infection. Nat Med.

[18] Focosi D, Maggi F, Casadevall A. Mucosal vaccines, sterilizing immunity, and the future of SARS-CoV-2 virulence. Viruses. 2022;14(2). 10.3390/v14020187 PMC8878800 35215783

[19] Dan JM, Mateus J, Kato Y, Hastie KM, Yu ED, Faliti CE et al. Immunological memory to SARS-CoV-2 assessed for up to 8 months after infection. Science. 2021;371(6529). 10.1126/science.abf4063 PMC7919858 33408181

